# De Novo Transcriptomic Profiling of *Piper chaba*: Elucidating the Genetic Basis of Piperine Biosynthesis and Metabolic Pathways

**DOI:** 10.1155/ijog/7117420

**Published:** 2026-06-27

**Authors:** Deeksha Singh, Shivangi Mathur, Rajiv Ranjan

**Affiliations:** ^1^ Plant Biotechnology Lab, Department of Botany, Faculty of Science, Dayalbagh Educational Institute (Deemed to Be University), Agra, Uttar Pradesh, India

**Keywords:** de novo transcriptome, differential gene expression, *Piper chaba*, piperine biosynthesis, SSR, transcription factors

## Abstract

Medicinal plants serve as invaluable sources of bioactive compounds, yet the molecular basis of their secondary metabolite biosynthesis remains largely unexplored. *Piper chaba* Hunter, an important but understudied member of the Piperaceae family, is known for its pharmacologically active alkaloids, particularly piperine. To our knowledge, this is the first de novo transcriptomic profiling of spike, leaf, and root tissues of *P. chaba* to uncover the genetic pathways regulating its metabolite production. Piperine, a major bioactive compound, was quantified using UPLC. The highest concentration was observed in the spike (331.3 mg/g), followed by the root (10.3 mg/g) and leaves (2.82 mg/g). High‐quality RNA sequencing of leaves, roots, and spikes using next‐generation sequencing (NGS) generated 228,481 transcripts, and 184,574 unigenes were identified after redundancy removal. Coding sequences (CDSs) derived from these unigenes were annotated using BLASTX and KEGG databases, which highlighted significant metabolic pathways, including those related to piperine biosynthesis. Thirteen candidate genes potentially associated with the piperine biosynthetic pathway were identified based on transcriptome annotation and pathway analysis. Validation of nine selected genes, including farnesyl pyrophosphate synthase and piperic acid synthase, was performed through qRT‐PCR using the 2^−*ΔΔ*Ct^ method, supporting their expression patterns potentially associated with piperine and related metabolite biosynthesis. Functional annotation categorized the CDS into Gene Ontology domains, with transcription factors such as bHLH and NAC families playing prominent roles in metabolic regulation. Additionally, 5050 SSRs were identified, offering potential markers for genetic studies. This pioneering study establishes a molecular framework for understanding the biosynthetic pathways of *P. chaba*, providing valuable insights for its application in sustainable medicine and agriculture.

## 1. Introduction

Herbal medicines have been essential to human health and survival since ancient times, serving as the foundation of early healthcare systems [1]. The transition from conventional medicine to modern pharmacology has primarily been accelerated by the investigation of medicinal plants and their phytoconstituents. Among these, the genus *Piper* is of particular significance within the family Piperaceae, comprising approximately 1000 species found in tropical and subtropical regions [2]. Several species within this genus, including *Piper nigrum* (black pepper), *Piper betle* (betel leaf), and *Piper longum* (long pepper), have been extensively studied for their bioactive constituents and therapeutic applications.

Beyond these well‐known species, *Piper chaba* Hunter represents a lesser explored yet economically and pharmacologically significant species [3]. *P. chaba* is a perennial flowering vine belonging to the family Piperaceae. It is indigenous to South and Southeast Asia and is commonly known as Badi Pippali or Bangla Tipali in India [4].

Morphologically, *P. chaba* is a creeping vine that grows along the ground and can also climb on trees. The plant bears oval leaves measuring approximately 3–5 inches in length. The flowers are monoecious and zygomorphic and predominantly bloom during the monsoon season. The fruits are elongated and can reach up to 3 inches in length. They turn red when ripe and become dark brown or black upon drying [5, 6].

Traditional medicinal applications of *P. chaba* primarily involve the use of its stem, root, and fruit. These plant parts contain several bioactive compounds, including alkaloids such as piperine, piperanine, pipernonaline, piperamine, 2,4‐decadienoic acid piperidide, kusunokinin, and pellitorine [7]. The root possesses alexiteric properties and has been traditionally used in the treatment of asthma, bronchitis, and consumption. The fruit exhibits stimulating and carminative properties and is commonly used to alleviate hemorrhoidal symptoms [8]. The stem is traditionally used to relieve postdelivery discomfort in mothers and to treat rheumatic pain and diarrhea [9]. The edible swollen stem is also consumed for its effectiveness in alleviating colds and coughs and in enhancing immunity against respiratory ailments [10].

Pharmacological studies have demonstrated that *P. chaba* possesses antibacterial, carminative, expectorant, analgesic, hypotensive, and smooth muscle relaxant properties. Piperine, a major alkaloid present in *P. chaba* and other species of the *Piper* genus, exhibits a wide range of pharmacological activities. These include antibacterial, immunomodulatory, antimutagenic, hepatoprotective, antioxidant, antimetastatic, and anticancer properties. Another compound, chabamide, a dimeric amide alkaloid isolated from the stem of *P. chaba*, has been reported to exhibit antimalarial, antituberculosis, and cytotoxic activities [11, 12, 13]. Similarly, piplartine, an alkaloid amide derived from this plant, has also been identified as a promising anticancer compound [14, 15].

Despite its extensive ethnobotanical and pharmacological significance, the molecular mechanisms underlying the biosynthesis of its bioactive compounds remain largely unexplored. Transcriptomic studies provide important insights into gene expression patterns and regulatory pathways involved in secondary metabolite biosynthesis. Such studies also provide a foundation for future biotechnological and pharmaceutical applications [16–18]. However, no transcriptomic studies have yet been reported for *P. chaba*, leaving a significant gap in scientific knowledge.

To address this gap, the present study is aimed at performing the first transcriptomic analysis of *P. chaba*. The study focuses on identifying key genes and pathways associated with the biosynthesis of its bioactive compounds. This research will improve the scientific understanding of *P. chaba* and support its sustainable utilization in medicine, agriculture, and biopharmaceutical industries. Furthermore, the findings will provide a valuable genomic resource for future studies on metabolic engineering, conservation, and bioprospecting of this important medicinal plant.

## 2. Materials and Methods

In January 2023, fresh and healthy plant samples of *P. chaba*, including roots, leaves, and spikes, were collected from the Herbal Garden of Dayalbagh Educational Institute (DEI), Dayalbagh, Agra. The freshly collected healthy plant samples were carefully rinsed first with groundwater and, subsequently, utilized distilled water to remove any adhering dirt. The samples were then air‐dried in a shaded area at 25°C for 12–15 days to preserve the active compounds. Afterward, the dried plant materials were pulverized with a machine grinder. The pulverized samples were subjected to sieving using a 2 mm mesh sieve.

### 2.1. Quantitative Analysis of Piperine by UPLC

The 5 g of powdered *P. chaba* roots, leaves, and spikes was refluxed individually in 50 mL of methanol for 6 h at a steady temperature. Samples were concentrated using a rotary vacuum evaporator. A final concentration of 10 mg/mL was obtained by dissolving 10 mg of each sample in 1 mL of methanol. Following reconstitution, an aliquot of the solution was filtered using a 0.22‐*μ*m filter.

A precisely measured amount (10 mg) of piperine (CAS No. 94‐62‐2) was added to methanol (10 mL) to make a stock solution of 1 mg/mL. The solution was degassed in a sonicator for 10 min and subsequently filtered using a 0.22‐*μ*m nylon membrane before dilution preparation (working solution). A standard solution of piperine (1 mg/mL) was diluted into five distinct concentrations (25, 50, 100, 150, and 200 *μ*g/mL). These solutions were subjected to the UPLC System for the preparation of a calibration graph. A calibration curve was formed by correlating the peak area with the piperine concentrations. The correlation coefficient consistently exceeded 0.99 in all analyses [19].

Analysis was done using an ACQUITY UPLC H‐Class System (Waters, Milford, United States) with the quaternary solvent manager and PDA detector and a Hypersil GOLD C18 column (2.1 × 100 mm, 1.9 mm particle size) (ACQUITY UPLC, Waters, Milford, United States) maintained at 25°C. Formic acid (0.1%) in water (Buffer A) and formic acid (0.1%) in methanol (Buffer B) were utilized as mobile phases. A flow rate of 0.200 mL/min was used, and a sample volume of 3 *μ*L was injected. Observations at 343 nm were made simultaneously. Piperine was quantified using a calibration curve (*R* ^2^ ≥ 0.99) in the 25–200 *μ*g/mL range for all standards. The chromatogram was documented, and the piperine peak was identified based on the retention time obtained from the standard solutions. The peak area of piperine in the sample extract was integrated and analyzed by comparing the calibration curve to evaluate the concentration. Software Empower 3 (Waters, Milford, United States) was utilized for processing chromatography data.

### 2.2. RNA Sequencing and Transcriptome Analysis of *P*. *chaba*

#### 2.2.1. Plant Material

**Figure 1:**
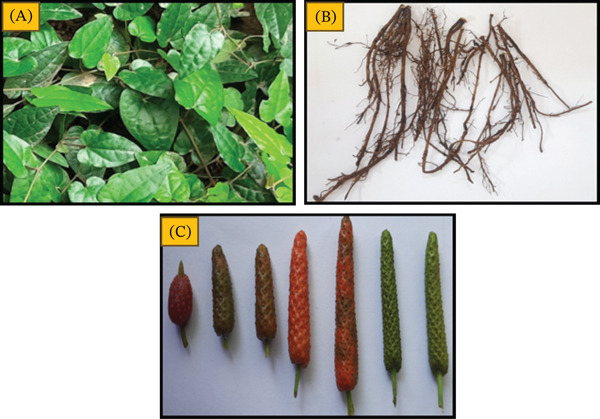
*Piper chaba* plant parts used in the analysis. (A) Leaves, (B) roots, and (C) spikes.

In January 2023, fresh and healthy plant samples of *P. chaba* were collected from the Herbal Garden of DEI, Dayalbagh, Agra, under natural field conditions (average temperature 15°C–22°C, relative humidity 50%–60%). Three biological replicates were collected for each tissue type including young leaves, roots, and spikes (Figure 1) from different healthy plants of the same age and growth stage. All samples were excised using a sterilized blade, immediately frozen in liquid nitrogen in the field, and stored at −80°C until further use to preserve RNA and metabolite integrity.

#### 2.2.2. RNA Isolation and Library Construction

Total RNA was isolated independently from three biological replicates of each tissue (leaves, roots, and spikes) using the Alexgen Total RNA Extraction Kit. Equal quantities of RNA from the three biological replicates were pooled for each tissue prior to library preparation to generate a representative transcriptome dataset. As a result, the RNA‐Seq analysis in this study represents expression profiling rather than statistically validated differential gene expression analysis. Accordingly, three tissue‐specific RNA‐Seq libraries representing leaf, root, and spike tissues were sequenced following a pooling strategy previously described in similar transcriptomic studies [20, 21]. The paired‐end sequencing libraries were constructed utilizing the KAPA mRNA HyperPrep Kit for Illumina as per the specified methodology. RNA was quantified using a Qubit 4.0 Fluorometer, while its quality was assessed by 1.2% agarose gel electrophoresis. The library preparation procedure started with an input of 500–1000 ng total RNA. mRNA enrichment was performed as per the user manual, followed by fragmentation of mRNA, synthesis of first‐ and second‐strand cDNA, end‐repair, 3 ^′^ adenylation, adapter ligation, selective enrichment of adapter‐ligated DNA fragments through PCR amplification, and subsequent validation of the library on the Agilent 4150 TapeStation. The completed library was combined with other samples, denatured, and loaded onto the flow cell. Cluster generation and sequencing were conducted using the Illumina NovaSeq 6000 platform to produce 2 × 150 bp paired‐end reads. The amplified libraries were examined on a TapeStation 4150 (Agilent Technologies) utilizing HSD100 ScreenTape.

#### 2.2.3. De Novo Transcriptome Assembly (Master Assembly)

High‐quality adapter‐trimmed reads from all *P. chaba* samples were generated by first assessing raw read quality using FastQC, followed by adapter and quality trimming with Trim Galore (Version 0.6.4). The resulting clean reads were then combined to form a master/combined assembly using Trinity (Version 2.14.0) with default parameters (*k*‐mer size of 25). This produced a common assembly for the subsequent annotation of transcripts and investigation of differential expression across samples. The completeness of the *P. chaba* transcriptome was assessed using BUSCO analysis against the Viridiplantae_odb10 database (updated on December 5, 2024). Default parameters were applied to classify orthologs as complete, duplicated, fragmented, or missing, ensuring a standardized evaluation of transcriptome quality for downstream functional and comparative analyses [22].

Transcripts of combined assembly undergo additional processing for unigene prediction using the CD‐HIT package (Version 4.8.1). The CD‐HIT‐EST program was employed to eliminate shorter redundant transcripts that were completely covered by other transcripts showing > 90% identity. The resultant nonredundant (NR) clustered transcripts were then categorized as unigenes. Coding sequence (CDS) was anticipated from the unigene sequences utilizing TransDecoder software (Version 5.6.0) with default settings, indicating a minimum encoded protein length of 60 amino acids (AAs). All CDSs were searched against the transcription factor (TF) database (http://planttfdb.cbi.pku.edu.cn/download.php) using BLASTX with an *e*‐value threshold of 1e − 5 for the identification of TF families. Gene expression levels were normalized using counts per million (CPM) to facilitate comparison of relative transcript abundance across tissues at default parameters.

### 2.3. Quantitative Gene Expression Analysis of *P. chaba*

This study focused on the quantitative gene expression analysis of *P. chaba*, particularly genes involved in the piperine biosynthesis pathway. RNA was isolated using the PureLink RNA Mini Kit and processed following standard protocols to ensure purity and integrity, which was confirmed via gel electrophoresis. First‐strand cDNA was synthesized using the R2D 1st strand cDNA synthesis kit, with random or gene‐specific primers. Primers for target genes, selected based on transcriptome expression patterns, were designed using the PrimerQuest tool and optimized for qRT‐PCR analysis.

A total of nine genes, including an internal control (Ubiquitin C [UbC]), were analyzed across leaves, roots, and spikes of *P. chaba* using the Brilliant III Ultra‐Fast SYBR Green QPCR kit. The qRT‐PCR reactions were conducted in 96‐well plates under thermocycler conditions that included an initial denaturation at 95°C for 10 min, followed by 40 amplification cycles at 95°C for 10 s, 53°C for 45 s, and 72°C for 1 min, concluding with a melt curve analysis at 95°C for 30 s, 53°C for 30 s, and 30 s at 95°C. Relative gene expression levels were calculated using the comparative 2^−*ΔΔ*Ct^ method. The Ct values of the target genes were first normalized with the internal reference gene UbC to obtain *Δ*Ct values (*Δ*Ct = Ct target − Ct reference). The *ΔΔ*Ct values were then calculated by comparing the *Δ*Ct of each sample with the *Δ*Ct of the control sample (root tissue). Fold change in gene expression was calculated as 2^−*ΔΔ*Ct^.

Data from three biological replicates were statistically analyzed to identify significant expression patterns, enhancing the understanding of secondary metabolite biosynthesis in *P. chaba*. Genes with an absolute log_2_ fold change ≥ 1 were considered genes showing notable expression differences between tissues.

### 2.4. Functional Annotation by Gene Ontology (GO) and Kyoto Encyclopedia of Genes and Genomes (KEGG)

Functional annotation of the predicted CDSs of *P. chaba* was conducted by aligning them to NR protein databases from the National Center for Biotechnology Information (NCBI) using BLASTX, with a minimum *e*‐value threshold of 1e − 5. BLAST 2.3.13+ software was employed for independent functional annotation and classification. All CDSs exhibited BLASTX hits against the NR database on NCBI. Predicted CDS was also searched against KOG, Pfam, and UniProt databases using BLASTX. The optimal match output from each BLAST was utilized for annotating the CDS. After each BLAST search, annotation tags, irrespective of hits and including those with predicted annotations, were extracted from the following sequential BLAST search. The expected functions of CDS/proteins were elucidated through the use of GO assignments. GO mappings provide a set of descriptive terms that characterize the qualities of gene products. The terms were categorized into three main domains: biological process (BP), molecular function (MF), and cellular component (CC). GO mapping was conducted to obtain GO terms for all functionally annotated CDSs from BLASTX against the NR database using OmicsBox (Version 3.0).

The KEGG Automatic Annotation Server (KAAS) (http://www.genome.jp/kegg/ko.html) was utilized to functionally annotate the CDSs through BLAST comparisons with the KEGG gene database [23–25]. The BBH (bidirectional best hit) method was utilized to assign KEGG Orthology (KO) terms. The KO database was utilized for pathway mapping. The CDSs were assignments of polypeptides produced by the combined assembly and mapped onto metabolic pathways according to KEGG.

### 2.5. Phylogenetic Analysis

A phylogenetic tree of the 13 piperine biosynthetic pathway genes was constructed using MEGA 12. The tree was generated using the neighbor‐joining method with 1000 bootstrap replicates.

### 2.6. Statistics

The statistical significance of gene expression differences was determined using one‐way analysis of variance (ANOVA), followed by Duncan′s multiple range test (DMRT) as a post hoc test for multiple comparisons.

## 3. Results

### 3.1. UPLC‐Based Estimation of Piperine Content in Different Parts of the Plant

The piperine content of *P. chaba* samples including roots, leaves, and spikes was quantified using a UPLC method. Piperine concentrations were determined using the calibration curve. The calibration curve demonstrated that the computed piperine concentrations closely align with the anticipated values, signifying a strong correlation with negligible variance. All samples exhibited a predominant peak corresponding to piperine, as determined by an authentic piperine standard. A standard curve was established (Supporting Information 8: Figure S1) utilizing different concentrations of piperine (25–200 *μ*g/mL), resulting in a correlation coefficient of 0.998556.

**Figure 2:**
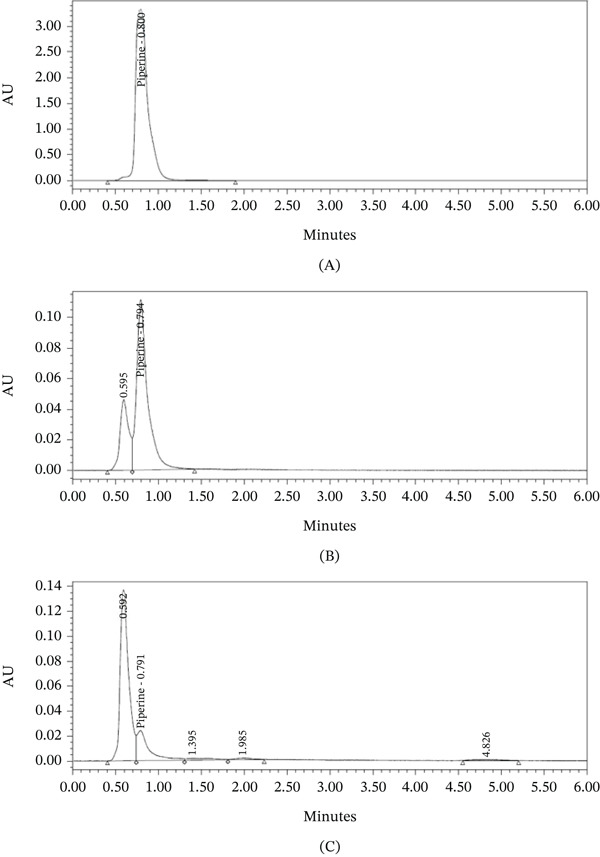
UPLC‐PDA chromatograms showing the detection of piperine in different tissues of *Piper chaba*. (A) Spikes, (B) roots, and (C) leaves. The *x*‐axis represents retention time (minutes), while the *y*‐axis indicates absorbance (AU).

The quantification of piperine in various parts of *P. chaba* is presented in Table 1. The UPLC chromatograms of all samples, depicted in Figure 2, exhibited a consistent piperine profile throughout. Piperine was identified as the dominant peak in the spike extract, representing 100% of the integrated chromatographic area under piperine‐specific detection at 343 nm and a concentration of 331.3 mg/g. The retention time of piperine in the root is 0.794 min, with 10.3 mg/g of piperine accounting for 75.06% of the area. The leaves exhibited minimal piperine concentration, with a retention time of 0.791 min, encompassing 19.17% of the area and with a total 2.82 mg/g piperine content.

**Table 1:** UPLC‐based quantification of piperine in different plant parts of *Piper chaba.*

S. no.	Plant part	Analyte	Retention time (min)	Formula	% Area	Amount (mg/g)
1.	Spike	Piperine	0.800	C_17_H_19_NO_3_	100.00	331.3
2.	Root	Piperine	0.794	C_17_H_19_NO_3_	75.06	10.3
3.	Leaves	Piperine	0.791	C_17_H_19_NO_3_	19.17	2.82

### 3.2. Transcriptome Sequencing and De Novo Assembly

Next‐generation sequencing (NGS) of *P. chaba* leaf, spike, and root tissues was performed using the Illumina NovaSeq 6000 platform, generating high‐quality 2 × 150 bp paired‐end reads. The sequencing produced 423,985,512, 663,405,588, and 703,265,582 paired reads from leaf, spike, and root samples, respectively. De novo assembly of the high‐quality adapter‐trimmed reads generated 228,481 transcripts with a total transcriptome size of 25,068,437 bases (Table 2). The average, maximum, and minimum transcript lengths were 1097, 19,061, and 300 bp, respectively.

**Table 2:** De novo transcriptome assembly statistics of *Piper chaba.*

Description	De novo assembly statistics
Total no. of transcripts	228,481
Total transcriptome size (bp)	250,684,374
Average transcript length (bp)	1097
Maximum transcript length (bp)	19,061
Minimum transcript length (bp)	300
Transcript N50 (bp)	1664

The completeness of the transcriptome assembly was assessed using BUSCO analysis. Results indicated a high level of completeness, with 94.3% complete BUSCO genes, including 8.2% single copy and 86.1% duplicated genes. Additionally, 5.4% were fragmented, and only 0.3% were missing (Supporting Information 1: Table S1). These results indicate that the assembled transcriptome is of high quality and suitable for downstream functional and comparative genomic analyses.

### 3.3. Unigene Prediction From Master Assembly

The transcripts underwent additional processing for unigene prediction utilizing the CD‐HIT package. The CD‐HIT‐EST program was employed to eliminate shorter redundant transcripts that were entirely encompassed by other transcripts exhibiting > 90% identity. The resultant NR clustered transcripts were then classified as unigenes. A total of 184,574 unigenes were predicted from the master assembly. The total size of unigenes was 19,147,507 bases. The average, maximum, and shortest lengths of unigenes in the libraries were 1037, 19,061, and 300 bases, respectively.

### 3.4. CDS Obtained From Unigenes

CDSs were derived from the unigene sequences utilizing TransDecoder with default settings, establishing a minimum encoded protein length of 60 AAs. This approach identified the segments of unigenes that encode functional proteins, facilitating gene identification and functional annotation. A total of 94,453 CDS nucleotides and a corresponding quantity of CDS proteins were obtained from the identified unigenes (Supporting Information 2: Table S2). The cumulative size of the CDS nucleotide sequence was 70,981,896 bases, whereas the CDS protein comprised 23,660,632 AAs. The average, maximum, and minimum lengths of CDS nucleotides were 752, 13,098, and 255 bases, respectively. The subsequent distribution of CDS was conducted based on its length, and the data is illustrated in Supporting Information 9: Figure S2.

### 3.5. Function Annotation of Predicted CDS

**Figure 3:**
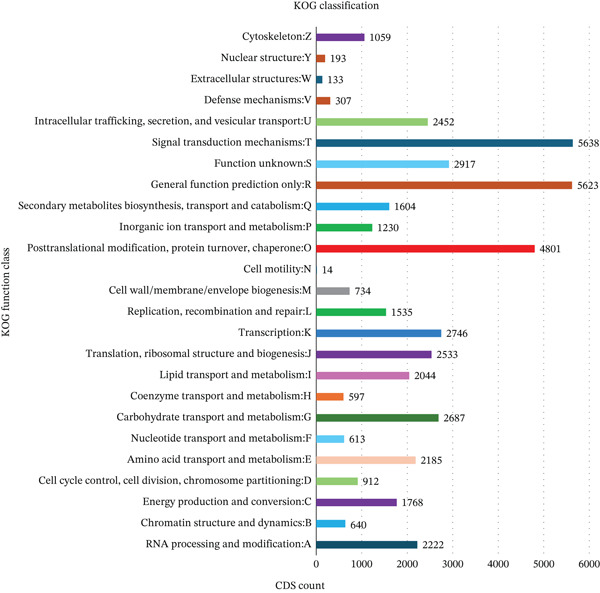
Gene Ontology (GO) functional classification of predicted *Piper chaba* coding sequences (CDSs). The *y*‐axis represents the GO functional terms, while the *x*‐axis indicates the number of CDS associated with each category.

Functional annotation of the predicted CDSs from the unigenes of three distinct tissues of *P. chaba* was performed through the BLASTX algorithm with an *e*‐value cutoff of 1e − 5. The annotation was conducted on the predicted CDS of the *P. chaba* sample by aligning it to the NR protein database of NCBI using BLASTX, with an *e*‐value threshold of less than 1e − 5. The BLASTX analysis statistics for the predicted CDS indicate 80,328 and 14,125 with BLAST hits and without BLAST hits from a total of 94,453 CDS sequences, shown in Supporting Information 3: Table S3. The species distribution assessment revealed that the predominant hits were associated with *Aristolochia fimbriata*, followed by *Cinnamomum micranthum* (Supporting Information 10: Figure S3). All CDSs were concurrently analyzed for similarity against UniProt, KOG, and Pfam utilizing BLASTX with an *e*‐value cutoff of 1e − 5. The outcomes of the similarity search across all databases are shown in Supporting Information 4: Table S4. KOG classification is an approach for categorizing CDS into functional categories based on orthologous relationships. The KOG analysis demonstrated that the most enriched KOG categories in *P. chaba* were “signal transduction mechanisms (T),” “general function prediction only (R),” and “posttranslational modification, protein turnover, chaperones (O)” (Figure 3). Pfam analysis is an approach utilized to detect and categorize protein domains and families within a set of sequences, including transcripts or genes. In the Pfam analysis, the predominant domains discovered in *P. chaba* were “Pkinase Tyr: protein tyrosine kinase,” followed by the “PPR_2 domain” (Supporting Information 11: Figure S4).

A Venn diagram is a visual tool employed to illustrate relationships among several sets of data. It illustrated the similarities and differences among the evaluated databases using overlapping circles. In Supporting Information 12: Figure S5, the Venn diagram illustrates that 28,127 CDSs were distributed throughout all four databases.

### 3.6. GO Sequence Distribution of NR Annotated CDS

A total of 15,458 CDSs were assigned at least one GO term, indicating that a single CDS may possess several GO terms. The GO category is allocated as follows: 10,236 CDSs were designated for BP, 8195 CDSs for CCs, and 12,139 CDSs for MF.

### 3.7. TF Identification

**Figure 4:**
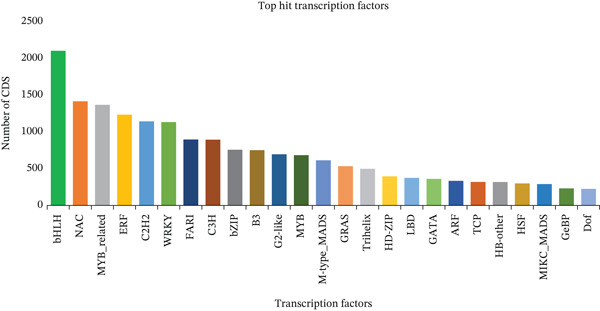
Distribution of transcription factor (TF) families identified from the predicted coding sequences (CDSs) of *Piper chaba*. The *x*‐axis represents the TF families, while the *y*‐axis indicates the number of TFs identified in each family.

TFs are essential for the biosynthesis of secondary metabolites. All anticipated CDSs were evaluated for similarity against the plant TF database utilizing BLASTX, with an *e*‐value threshold of 1e − 5. Among the 58 total hits, the most prevalent TF discovered was bHLH (basic helix–loop–helix), followed by the NAC family. Figure 4 illustrates the transcripts categorized by main TF families.

### 3.8. Functional Characterization and Pathway Analysis of CDS Using KEGG

**Figure 5:**
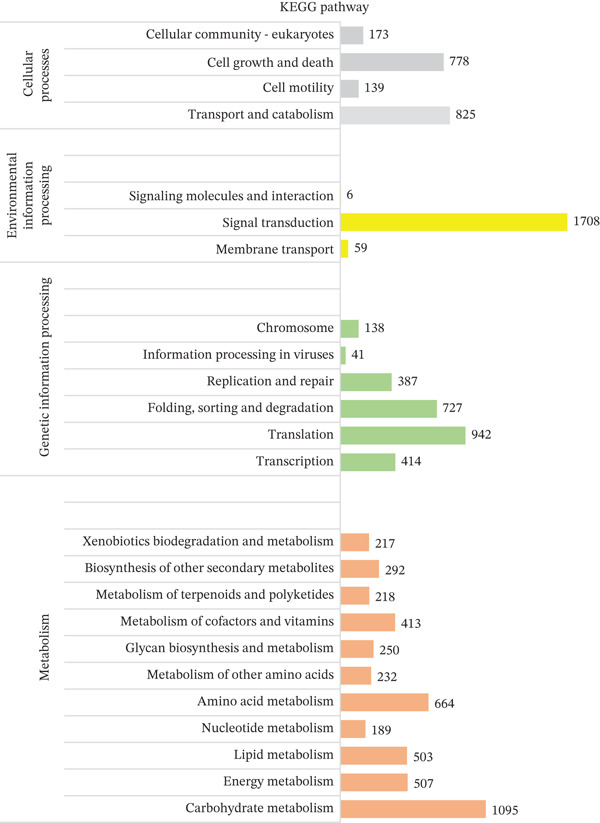
Kyoto Encyclopedia of Genes and Genomes (KEGG) pathway‐based functional annotation and classification of predicted *Piper chaba* coding sequences (CDSs). Different colors represent these major KEGG functional groups, and the length of each bar indicates the number of CDS assigned to the respective pathway.

Ortholog assignment and the mapping of the CDSs to biological pathways were conducted utilizing the KAAS. All the CDSs were evaluated against the KEGG database utilizing BLASTX with a threshold bit‐score value of 60 (default). Of the total 94,453 CDSs, 5876 were aligned with the KEGG database, indicating metabolic pathways of essential macromolecules like carbohydrates, lipids, cofactors, vitamins, AAs, nucleotides, terpenoids, and polyketides. Genetic information processing includes transcription, translation, folding, sorting, degradation, replication, repair, and information processing in viruses. Environmental information processing includes membrane transport, signal transduction, signaling molecules, and their interactions. The transcripts also indicated the genes associated with metabolism, environmental information processing, genetic information processing, and cellular functions, as illustrated in Figure 5.

### 3.9. Piperine Biosynthetic Pathway‐Related Genes

**Figure 6:**
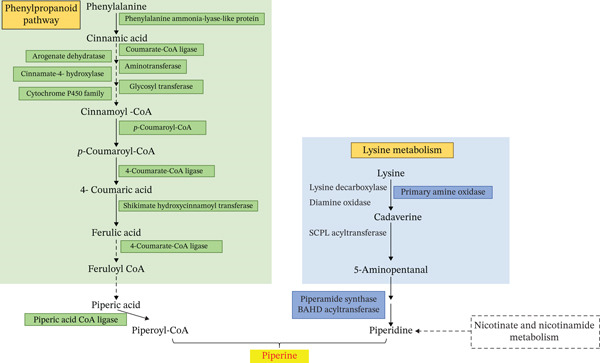
A schematic representation of the key genes encoding enzymes putatively involved in the piperine biosynthesis pathway, as identified through the transcriptomic analysis of *Piper chaba*. Genes detected in this study are highlighted within green and blue boxes, while dashed arrows indicate multistep enzymatic processes. The identified genes include phenylalanine ammonia‐lyase–like protein (*Unigene_64981_CDS_71273*), coumarate‐CoA ligase (*Unigene_51783_CDS_64888*), arogenate dehydratase (*Unigene_101207_CDS_576*), cinnamate 4‐hydroxylase (*Unigene_160753_CDS_38358*), aminotransferase (*Unigene_104210_CDS_2524*), Cytochrome P450 family (*Unigene_113463_CDS_9672*), glycosyl transferase (*Unigene_100256_CDS_86*), *p*‐coumaroyl‐CoA (*Unigene_30201_CDS_50615*), 4‐coumarate‐CoA ligase (*Unigene_51783_CDS_64887*), shikimate hydroxycinnamoyl transferase (*Unigene_16628_CDS_39877*), piperic acid CoA ligase (*Unigene_140631_CDS_26957*), primary amine oxidase (*Unigene_154287_CDS_34411*), and piperamide synthase/BADH acyltransferase (*Unigene_147284_CDS_30376*).

In the piperine biosynthetic pathway of *P. chaba*, the 13 genes encoding enzymes were identified including phenylalanine ammonia‐lyase–like protein (Unigene_64981_CDS_71273), coumarate‐CoA ligase (Unigene_51783_CDS_64888), arogenate dehydratase (Unigene_101207_CDS_576), cinnamate 4‐hydroxylase (Unigene_160753_CDS_38358), aminotransferase (Unigene_104210_CDS_2524), Cytochrome P450 (CYP) family (Unigene_113463_CDS_9672), glycosyl transferase (Unigene_100256_CDS_86), *p*‐coumaroyl‐CoA (Unigene_30201_CDS_50615), 4‐coumarate‐CoA ligase (4CL) (Unigene_51783_CDS_64887), shikimate hydroxycinnamoyl transferase (Unigene_16628_CDS_39877), piperic acid CoA ligase (Unigene_140631_CDS_26957), primary amine oxidase (Unigene_154287_CDS_34411), and piperamide synthase/BADH acyltransferase (Unigene_147284_CDS_30376). Among the total identified genes, some were associated with lysine metabolism, while others were associated with the phenylpropanoid pathway (Figure 6).

### 3.10. Gene Expression Analysis and Validation

To investigate gene expression related to piperine biosynthesis, key genes were selected based on their involvement in relevant MFs and BPs. UbC was used as an internal control, while eight target genes, including farnesyl pyrophosphate synthase (FPPS), serine–glyoxylate aminotransferase (SGAT), UDP‐glycosyltransferase (UGT), CYP, phytoene synthase (PSY), piperic acid synthase (PAS), 4CL, and glycosyltransferase (GT), were analyzed. A comprehensive overview of these genes and their functions is provided in Table 3.

**Table 3:** Selected genes for validation through qRT‐PCR.

S. no.	CDS	Name of genes	Function of genes
1.	Unigene_78358_CDS_80420	Ubiquitin C	Internal control gene
2.	Unigene_88997_CDS_88054	Farnesyl pyrophosphate synthase	Key enzyme in the isoprenoid biosynthetic pathway
3.	Unigene_93637_CDS_90424	Serine–glyoxylate aminotransferase	Role in the serine assimilation pathway and photorespiration
4.	Unigene_17935_CDS_43258	UDP‐glycosyltransferase	Biosynthesis of natural compounds such as flavonoids and terpenoids
5.	Unigene_134546_CDS_22123	Cytochrome P450	Regulate the biosynthesis and catabolism of plant hormones like auxins, cytokinins, and gibberellins
6.	Unigene_73189_CDS_77315	Phytoene synthase	Carotenoid biosynthesis pathway
7.	Unigene_81681_CDS_82640	Piperic acid synthase	Biosynthetic pathway of piperine
8.	Unigene_51783_CDS_64887	Coumarate‐CoA ligase	Role in the biosynthesis of piperine by activating intermediate acids to the corresponding CoA
9.	Unigene_100256_CDS_86	Glycosyl transferase	Catalyzed the biosynthesis of complex carbohydrates

For primer design, the Integrated DNA Technologies (IDT) online tool was used to generate primers with optimized amplicon length, melting temperature, and GC content. Table 4 details the forward and reverse primer sequences used for gene validation. RNA was isolated using the PureLink RNA Mini Kit, followed by qualitative and quantitative analysis. RNA integrity was assessed by 1.2% agarose gel electrophoresis in TBE buffer (Supporting Information 13: Figure S6), and RNA concentration was determined using a NanoDrop spectrophotometer (Agilent Technologies NanoDrop 800), as summarized in Supporting Information 5: Table S5.

**Table 4:** qRT‐PCR primers for validation of selected genes in *Piper chaba.*

Target genes	Transcript ID	GC %	Forward/reverse	Primer sequence (5 ^′^to 3 ^′^)	Amplicon
Ubiquitin C (UbC)	Unigene_78358_CDS_80420 (housekeeping)	50	F	GATTGGGTGGTGGTCACTATAC	91
		45.455	R	CTGGCTGGAGAAACATGACTAT	
Farnesyl pyrophosphate synthase (FPPS)	Unigene_88997_CDS_88054	47.619	F	CAGGTGAGCGTTTGGATAGTT	96
		50	R	CGAAGCAGTCCAAGTAGTCATC	
Serine–glyoxylate aminotransferase (SGAT)	Unigene_93637_CDS_90424		F	GCGCCAATCTTGACATTCTTG	95
			R	ACCAGTTGCAGTCTCGTTATG	
UDP‐glycosyltransferase (UGT)	Unigene_17935_CDS_43258	50	F	GCCTCTTACCCACGAGATTATG	97
		50	R	TTGAAGGGTGAGCCAATACC	
Cytochrome P450 (CYP)	Unigene_134546_CDS_22123	50	F	AGAGGTTGTTGAGGGTTGTG	105
		50	R	CAGATGTGTTGGGTCGAAGT	
Phytoene synthase (PSY)	Unigene_73189_CDS_77315	45.455	F	AGACAAGTGGAGGAGCTTTATG	105
		47.826	R	GGCCATCTACTAGCTTGGTTAAG	
Piperic acid synthase (PAS)	Unigene_81681_CDS_82640	50	F	GGAACTTCACACTCCTGCTT	93
		45.455	R	ACATGGTCTCCGGTTTCATATC	
4‐Coumarate‐CoA ligase (4CL)	Unigene_51783_CDS_64887	47.368	F	TGCTGTTGTCCCGATGAAA	98
		45.455	R	GCTTGATCTCATCCTCGGTAAT	
Glycosyl transferase (GT)	Unigene_100256_CDS_86	50	F	CTGGAAAGTGTTGGTCCTCTAC	100
		47.619	R	CATCAGAAGTGTTCCCATCCA	

**Figure 7:**
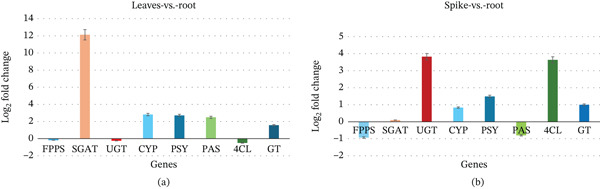
Expression profiles of selected genes validated by qRT‐PCR (quantitative real‐time polymerase chain reaction). (a) Leaf‐versus‐root comparison and (b) spike‐versus‐root comparison. Error bars represent mean ± standard deviation (SD) from three biological replicates.

Quantitative gene expression analysis was performed using qRT‐PCR to validate candidate genes identified from transcriptome expression profiling in the leaf‐versus‐root and spike‐versus‐root comparisons. qRT‐PCR reactions included a no‐template control, and UbC was used as the internal reference gene (Table 4). Relative transcript levels were calculated using the 2^−*ΔΔ*Ct^ method. Dissociation curve analysis confirmed the specificity of amplification and the absence of primer–dimer artifacts. Gene expression profiles (log_2_ fold change ± SE) are shown in Figure 7. The results indicate tissue‐specific expression of several genes potentially associated with piperine biosynthesis, with some genes showing higher expression in leaves, while others were predominantly expressed in spike tissues.

In the leaf‐versus‐root comparison, SGAT exhibited strong upregulation, with a log_2_ fold change greater than 11, indicating higher expression in leaves. Other genes, including CYP, PSY, PAS, and GT, showed moderate upregulation. In contrast, UGT and 4CL displayed negligible or slightly downregulated expression levels.

**Figure 8:**
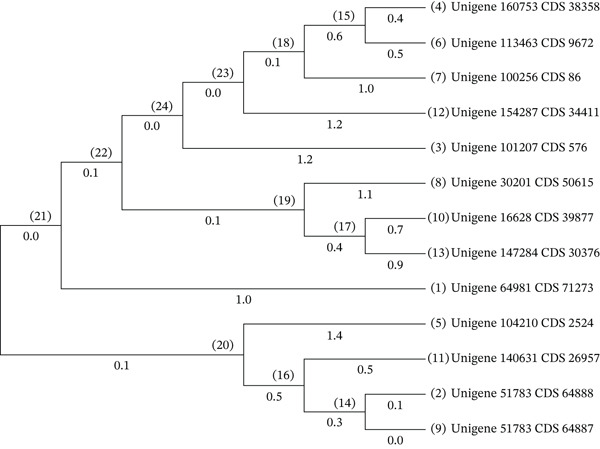
Phylogenetic analysis of 13 key genes involved in the piperine biosynthetic pathway.

In the spike‐versus‐root comparison, UGT and 4CL showed notable upregulation, with log_2_ fold changes greater than 3, indicating higher expression in spike tissue. SGAT, CYP, PSY, and GT also exhibited increased expression in spike tissues, whereas FPPS and PAS showed relatively low or slightly downregulated expression. UbC, used as the reference gene, showed stable expression across all tissues. The evolutionary relationships among these genes were further analyzed using phylogenetic tree construction (Figure 8).

The qRT‐PCR expression patterns were compared with RNA‐Seq expression values represented as CPM. A generally consistent expression trend was observed between both methods for most genes. For example, SGAT and PSY showed strong upregulation in the leaf‐versus‐root comparison, with qRT‐PCR log_2_ fold change values of 12.13 and 2.71, respectively, while the RNA‐Seq data also indicated relatively higher transcript abundance (CPM values of 6.47 and 4.55) (Table 5). Similarly, genes such as CYP, PAS, and PSY showed comparable expression trends in both datasets (Supporting Information 6: Table S6). In the spike‐versus‐root comparison, UGT and 4CL showed strong upregulation in qRT‐PCR analysis (3.82 and 3.64, respectively), while RNA‐Seq data also indicated higher transcript abundance for these genes (3.65 and 5.65 CPM). This general agreement between qRT‐PCR and RNA‐Seq expression patterns supports the reliability of the transcriptomic dataset (Supporting Information 7: Table S7).

**Table 5:** Log_2_ fold change in differential expression of selected genes.

S. no.	Gene	Log_2_ fold change (root vs. leaves)	Log_2_ fold change (root vs. spike)
1.	Ubiquitin C (UbC)	0	0
2.	Farnesyl pyrophosphate synthase (FPPS)	−0.18686	−0.93207
3.	Serine–glyoxylate aminotransferase (SGAT)	12.12573	0.098595
4.	UDP‐glycosyltransferase (UGT)	−0.25232	3.824167
5.	Cytochrome P450 (CYP)	2.815387	0.833674
6.	Phytoene synthase (PSY)	2.713209	1.487527
7.	Piperic acid synthase (PAS)	2.496661	−0.80129
8.	4‐Coumarate‐CoA ligase (4CL)	−0.50464	3.638918
9.	Glycosyl transferase (GT)	1.569168	0.999972

## 4. Discussion

Piperine was identified as the major secondary metabolite in *P. chaba* through UPLC analysis, with the highest concentration detected in the spike (331.3 mg/g), followed by the root (10.3 mg/g) and leaves (2.82 mg/g). These results demonstrate a clear organ‐specific distribution of piperine, with the spike acting as the primary reservoir. Similar patterns have been reported in other *Piper* species, where reproductive structures accumulate higher levels of specialized metabolites than vegetative tissues. For example, *P. nigrum* contains approximately 46.6 mg of piperine per gram of pepper [26], while *P. longum* fruits contain about 16.362 mg/g [27].

Previous transcriptomic investigations in *P. nigrum*, *P. longum*, and *Piper retrofractum* have explored gene expression in root, flower, and fruit tissues to obtain insights into piperine biosynthesis [28–34]. However, limited genomic information has been available for *P. chaba*, restricting molecular studies of its metabolic pathways. In the present study, transcriptome sequencing of root, leaf, and spike tissues was conducted to explore gene expression patterns potentially associated with piperine biosynthesis. Comparative analysis of tissue‐specific transcriptomes together with metabolite accumulation provides initial insights into the molecular basis of piperine production in this species.

The biosynthetic pathway of piperine remains only partially understood, and most functional studies have been performed in *P. nigrum* [35, 36]. Current evidence indicates that piperine is synthesized through the condensation of piperoyl‐CoA and piperidine, derived from phenylalanine‐ and lysine‐related metabolic pathways, respectively [37, 38]. The BAHD acyltransferase enzyme pipBAHD1 catalyzes this coupling reaction to produce piperine [39], while piperoyl‐CoA is believed to originate from piperic acid through the activity of piperoyl‐CoA ligase [40–43]. These studies provide a conceptual framework for interpreting candidate genes identified in the present transcriptomic dataset.

Transcriptome analysis revealed several candidate genes potentially associated with piperine biosynthesis, including phenylalanine ammonia‐lyase, cinnamate 4‐hydroxylase, 4CL, CYP enzymes, GTs, shikimate hydroxycinnamoyl transferase, piperic acid CoA ligase, primary amine oxidase, and BAHD acyltransferase. Many of these enzymes participate in phenylpropanoid metabolism and related biochemical pathways, suggesting potential involvement in the formation of piperine precursors.

Validation of selected genes using qRT‐PCR supported the RNA‐Seq expression patterns and revealed tissue‐specific variation in transcript abundance. Some genes, including SGAT, PSY, and FPPS, are not established components of the canonical piperine biosynthetic pathway and may instead contribute to broader metabolic processes or precursor supply pathways. Differences in gene expression across tissues therefore likely reflect variations in metabolic activity rather than direct evidence of pathway involvement. The elevated piperine content observed in spike tissue corresponded with increased expression of several phenylpropanoid‐related genes, suggesting a possible relationship between metabolic gene activity and piperine accumulation. For instance, 4CL showed strong upregulation in spike tissue compared with root tissue (log_2_ fold change = 4), whereas expression was lower in leaves (log_2_ fold change = −0.5). Because 4CL is a key enzyme in the phenylpropanoid pathway, higher expression in spikes may indicate enhanced precursor availability for downstream alkaloid biosynthesis. Nevertheless, additional biochemical and functional studies will be necessary to confirm its precise role in piperine formation.

The transcriptome dataset also revealed multiple TF families, including bHLH, MYB‐related, NAC, and WRKY, which are known regulators of plant development and secondary metabolism [44–46]. In other *Piper* species, TFs such as MYB, WD40, WRKY, and LIM have been implicated in phenylpropanoid pathway regulation and may influence the expression of genes such as PAL, C4H, and 4CL [47]. The presence of these regulatory elements in *P. chaba* suggests that similar transcriptional networks may contribute to tissue‐specific metabolic activity and secondary metabolite accumulation.

Overall, the integration of metabolite profiling and transcriptome analysis provides initial insights into the molecular basis of piperine biosynthesis in *P. chaba* [48]. However, the observed relationships between gene expression patterns and metabolite accumulation represent correlations rather than direct evidence of enzymatic function. Further functional and enzyme‐level studies will be required to confirm the specific roles of these candidate genes in the piperine biosynthetic pathway and to better understand the regulation of secondary metabolite production in this species.

## 5. Conclusion

Understanding the genetic basis of secondary metabolite biosynthesis is essential for exploring the medicinal potential of plants. In this study, a comprehensive transcriptomic analysis of *P. chaba* was conducted to investigate the molecular mechanisms associated with piperine biosynthesis. Metabolite profiling confirmed that piperine accumulation is highest in spike tissue, highlighting its pharmacological importance. Transcriptome sequencing and functional annotation revealed numerous genes associated with metabolic and secondary metabolite pathways, including candidate genes potentially involved in the piperine biosynthetic pathway. Gene expression analysis, supported by qRT‐PCR validation, suggested tissue‐specific expression patterns of several biosynthetic genes. In addition, multiple TF families, including bHLH and NAC, were identified, which may contribute to the regulation of metabolic processes in *P. chaba*.

Overall, this study establishes an important transcriptomic resource for *P. chaba* and provides insights into the potential molecular basis of piperine biosynthesis. These findings offer a foundation for future studies aimed at further investigating the regulatory mechanisms involved in piperine biosynthesis.

## Author Contributions

D.S.: conceptualization, methodology, and writing original draft; S.M.: review and editing; R.R.: data analysis, review and editing, and supervision. All authors reviewed the results and the manuscript.

## Funding

The author (D.S.) acknowledges the financial assistance from CSIR‐UGC, New Delhi, through the Junior and Senior Research Fellowships. The author (D.S.) received financial support from CSIR‐UGC, New Delhi, in the form of Junior and Senior Research Fellowships.

## Conflicts of Interest

The authors declare no conflicts of interest.

## Supporting information


**Supporting Information** Additional supporting information can be found online in the Supporting Information section. **Supplementary Material 1.** Table S1: BUSCO analysis for *Piper chaba* transcriptome assembly. **Supplementary Material 2.** Table S2: Statistical overview of predicted coding sequences (CDSs), including number, length, and size distribution. **Supplementary Material 3.** Table S3: BLAST analysis statistics against the nonredundant (NR) database. **Supplementary Material 4.** Table S4: BLAST annotation statistics across protein databases (UniProt, KOG, and Pfam). **Supplementary Material 5.** Table S5: RNA yield and purity assessment from leaf, spike, and root tissues of *Piper chaba*. **Supplementary Material 6.** Table S6: Comparison of qRT‐PCR and RNA‐Seq (CPM) expression values for selected genes in leaf versus root, including *p* values and FDR. **Supplementary Material 7.** Table S7: Comparison of qRT‐PCR and RNA‐Seq (CPM) expression values for selected genes in spike versus root, including *p* values and FDR. **Supplementary Material 8.** Figure S1: UPLC chromatogram of piperine standard. **Supplementary Material 9.** Figure S2: Length distribution of predicted CDSs and proteins. **Supplementary Material 10.** Figure S3: Top species distribution based on BLAST analysis. **Supplementary Material 11.** Figure S4: Top Pfam domains identified in coding sequences. **Supplementary Material 12.** Figure S5: Venn diagram showing common CDS across NR, UniProt, KOG, and Pfam databases. **Supporting Information 13.** Figure S6: RNA isolation from leaf, spike, and root tissues of *Piper chaba*.

## Data Availability

The transcriptomic data of *Piper chaba* have been deposited in the NCBI Sequence Read Archive (SRA) under BioProject ID PRJNA1097210. The BioSample accession numbers for spikes, roots, and leaves are SAMN40894061, SAMN40894052, and SAMN40893896, respectively, with corresponding SRA Accession Numbers SRR28673946, SRR28673947, and SRR28673948.
