# Supplementary material for: De Novo Transcriptomic Profiling of Piper chaba: Elucidating the Genetic Basis of Piperine Biosynthesis and Metabolic Pathways
Source: Int J Genomics. 2026 Jun 27;2026:7117420. doi: 10.1155/ijog/7117420 (PMC13309848; doi:10.1155/ijog/7117420)
Supplement: Supplementary file 1 — Supporting Information Additional supporting information can be found online in the Supporting Information section. Supplementary Material 1. Table S1: BUSCO analysis for Piper chaba transcriptome assembly. Supplementary Material 2. Table S2: Statistical overview of predicted coding sequences (CDSs), including number, length, and size distribution. Supplementary Material 3. Table S3: BLAST analysis statistics against the nonredundant (NR) database. Supplementary Material 4. Table S4: BLAST annotation statistics across protein databases (UniProt, KOG, and Pfam). Supplementary Material 5. Table S5: RNA yield and purity assessment from leaf, spike, and root tissues of Piper chaba. Supplementary Material 6. Table S6: Comparison of qRT‐PCR and RNA‐Seq (CPM) expression values for selected genes in leaf versus root, including p values and FDR. Supplementary Material 7. Table S7: Comparison of qRT‐PCR and RNA‐Seq (CPM) expression values for selected genes in spike versus root, including p values and FDR. Supplementary Material 8. Figure S1: UPLC chromatogram of piperine standard. Supplementary Material 9. Figure S2: Length distribution of predicted CDSs and proteins. Supplementary Material 10. Figure S3: Top species distribution based on BLAST analysis. Supplementary Material 11. Figure S4: Top Pfam domains identified in coding sequences. Supplementary Material 12. Figure S5: Venn diagram showing common CDS across NR, UniProt, KOG, and Pfam databases. Supporting Information 13. Figure S6: RNA isolation from leaf, spike, and root tissues of Piper chaba. [file IJOG-2026-7117420-s001.docx]

**Supplementary File**

**Table S1:** BUSCO analysis for *Piper chaba* transcriptome

| C:94.3% [S:8.2%, D:86.1%], F:5.4%, M:0.3%, n:425 | |
| --- | --- |
| 401 | Complete BUSCOs (C) |
| 35 | Complete and single-copy BUSCOs (S) |
| 366 | Complete and duplicated BUSCOs (D) |
| 23 | Fragmented BUSCOs (F) |
| 1 | Missing BUSCOs (M) |
| 425 | Total BUSCO groups searched |

**Table S2:** Detailed statistical overview of predicted Coding Sequences (CDS) in *Piper chaba*

| **Description** | **CDS Nucleotide** | **CDS Protein** |
| --- | --- | --- |
| Total no of CDS | 94,453 | 94,453 |
| Total CDS size (bp/aa) | 70,981,896 | 23,660,632 |
| Average CDS length (bp) | 752 | 251 |
| Maximum CDS length (bp) | 13,098 | 4,366 |
| Minimum CDS length (bp) | 255 | 85 |

**Table S3:** BLAST analysis statistics against the Non-Redundant (NR) database

| **Sample Name** | **Total CDS** | **CDS with Hits** | **CDS with No Hits** |
| --- | --- | --- | --- |
| Master CDS | 94453 | 80328 | 14125 |

**Table S4**: BLAST analysis statistics across protein databases

| **Database** | **Hits against database** |
| --- | --- |
| Total | 94453 |
| Nr | 80328 |
| UniProt | 68356 |
| KOG | 42167 |
| Pfam | 40187 |

| **Sample** | **260/280** | **Concentration (ng/µl)** |
| --- | --- | --- |
| *P. chaba* (Leaves) | 2.093 | 586.032 |
| *P. chaba* (Spike) | 2.099 | 644.874 |
| *P. chaba* (Root) | 2.123 | 157.705 |

**Table S5:** RNA yield and purity assessment from Leaves, Spike, and Root of *Piper chaba*

**Table S6:** Comparison of qRT-PCR and RNA-Seq (CPM) expression values for selected genes in leave vs root, with corresponding p-values and FDR from RNA-Seq analysis.

| **Target genes** | **Transcript Id** | **qRT-PCR value** | **CPM value** | **P-value** | **FDR** |
| --- | --- | --- | --- | --- | --- |
| Farnesyl pyrophosphate synthase (FPPS) | Unigene_88997_CDS_88054 | -0.18686 | 2.514046032 | 7.21788E-06 | 0.000493568 |
|  |  |  |  |  |  |
| Serine--glyoxylate aminotransferase (SGAT) | Unigene_93637_CDS_90424 | 12.12573 | 6.46638061 | 3.70815E-11 | 2.01E-08 |
|  |  |  |  |  |  |
| UDP-glycosyltransferase (UGT) | Unigene_17935_CDS_43258 | -0.25232 | 2.50957032 | 0.000111877 | 0.004365 |
|  |  |  |  |  |  |
| Cytochrome P450  (CYP) | Unigene_134546_CDS_22123 | 2.815387 | 2.82766467 | 0.031169706 | 0.244325 |
|  |  |  |  |  |  |
| Phytoene synthase  (PSY) | Unigene_73189_CDS_77315 | 2.713209 | 4.548106814 | 1.79694E-11 | 1.08E-08 |
|  |  |  |  |  |  |
| Piperic acid synthase (PAS) | Unigene_81681_CDS_82640 | 2.496661 | 3.537882325 | 0.000247301 | 0.00819 |
|  |  |  |  |  |  |
| 4-Coumarate--CoA ligase  (4CL) | Unigene_51783_CDS_64887 | -0.50464 | 5.654914208 | 0.416801014 | 0.793594 |
|  |  |  |  |  |  |
| Glycosyl transferase (GT) | Unigene_100256_CDS_86 | 1.569168 | 1.12541606 | 0.156049827 | 0.522842 |
|  |  |  |  |  |  |
|  |  |  |  |  |  |

**Table S7:** Comparison of qRT-PCR and RNA-Seq (CPM) expression values for selected genes in spike vs root, with corresponding p-values and FDR from RNA-Seq analysis.

| **Target genes** | **Transcript Id** | **qRT-PCR** | | **CPM value** | **P-value** | | **FDR** | |
| --- | --- | --- | --- | --- | --- | --- | --- | --- |
| Farnesyl pyrophosphate synthase (FPPS) | Unigene_88997_CDS_88054 | | -0.93207 | 3.260125452 | | 2.00947E-07 | | 2.33E-05 |
|  |  |  |  |  |  |  |  |  |
| Serine--glyoxylate aminotransferase (SGAT) | Unigene_93637_CDS_90424 | | 0.098595 | 4.696913853 | | 1.3311E-05 | | 0.00064 |
|  |  |  |  |  |  |  |  |  |
| UDP-glycosyltransferase (UGT) | Unigene_17935_CDS_43258 | | 3.824167 | 3.64871055 | | 2.78749E-07 | | 3.03E-05 |
|  |  |  |  |  |  |  |  |  |
| Cytochrome P450  (CYP) | Unigene_134546_CDS_22123 | | 0.833674 | 3.41765563 | | 0.003727938 | | 0.051897 |
|  |  |  |  |  |  |  |  |  |
| Phytoene synthase  (PSY) | Unigene_73189_CDS_77315 | | 1.487527 | 2.634839531 | | 1.57983E-05 | | 0.000738 |
|  |  |  |  |  |  |  |  |  |
| Piperic acid synthase (PAS) | Unigene_81681_CDS_82640 | | -0.80129 | 2.308953564 | | 0.319696167 | | 0.720205 |
|  |  |  |  |  |  |  |  |  |
| 4-Coumarate--CoA ligase  (4CL) | Unigene_51783_CDS_64887 | | 3.638918 | 5.654914208 | | 0.416801014 | | 0.793594 |
|  |  |  |  |  |  |  |  |  |
| Glycosyl transferase (GT) | Unigene_100256_CDS_86 | | 0.999972 | 1.12541606 | | 0.156049827 | | 0.522842 |
|  |  |  |  |  |  |  |  |  |

**
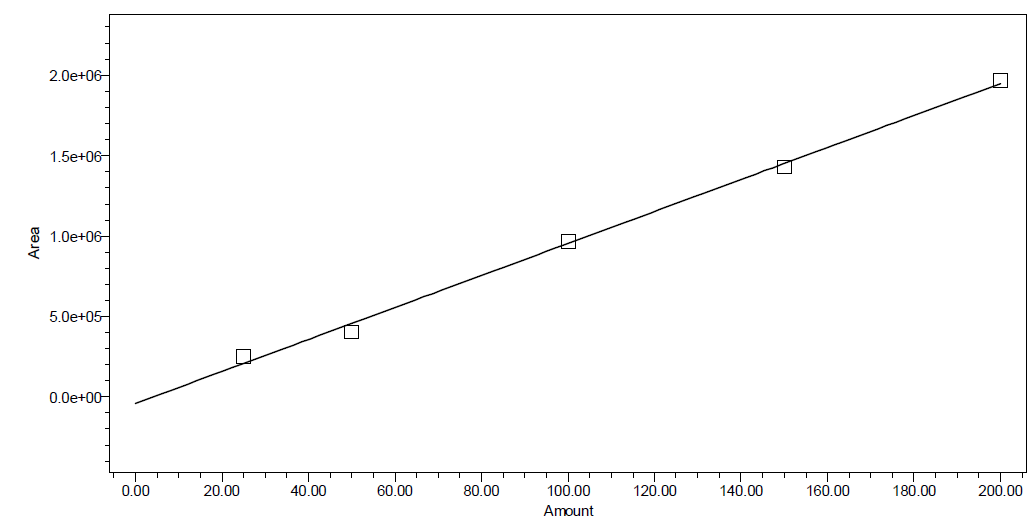
**

**Figure S1:** Chromatogram obtained from the UPLC with the methanolic extract of piperine standard where the x-axis represents the concentration values (µg/ml) and the y-axis represents the area under the plot.

**Figure S2:** Length distribution of predicted CDS and protein. The highest number of CDSs were observed in the length range of over 300 to 400 base pairs, followed by the length range of over 1000 to 5000 base pairs however the highest number of proteins is observed in the length range of over 200 base pairs, followed by the length range of over 200 to 300 base pairs.

**Figure S3**: Top species distribution of *Piper chaba* coding sequences (CDS) Based on BLAST hits.

**Figure S4:** Top Pfam domains identified in *Piper chaba* coding sequences (CDS).


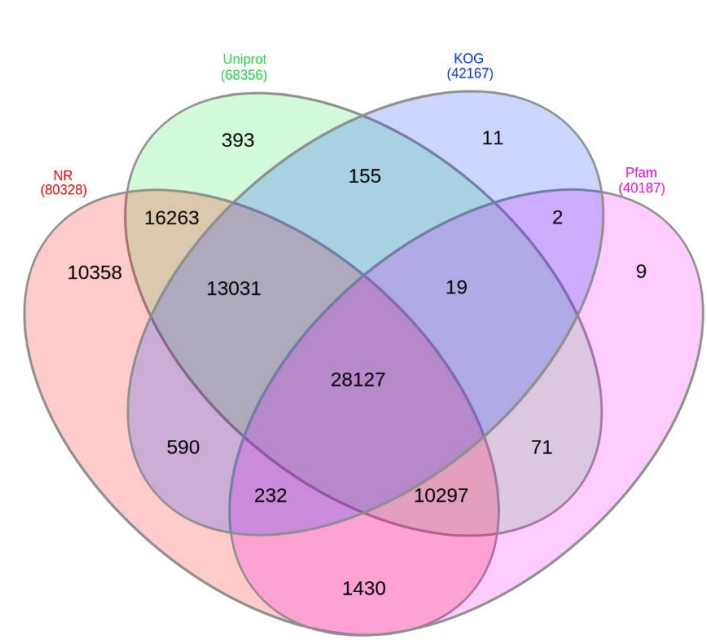


**Figure S5:** Venn diagram showing the common CDS in NR, Uniport, KOG, and Pfam databases


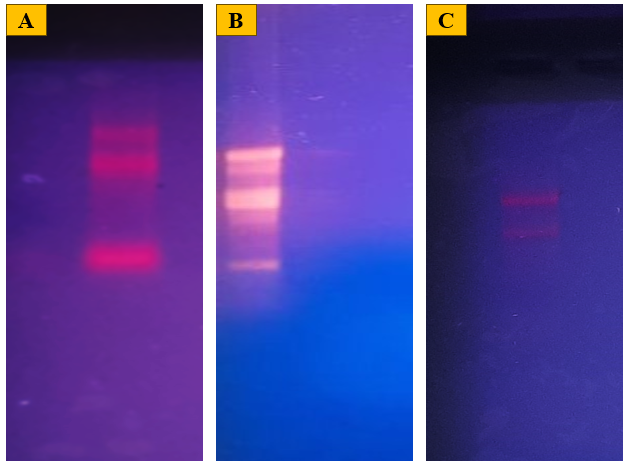


**Figure S6:** **A-** Isolated RNA of leaves from *P. chaba* **B-** Isolated RNA of the spike from *P. chaba* and **C-** Isolated RNA of root from *P. chaba.*
